# The epidemiology of road traffic accidents and associated factors among drivers in Dilla Town, Southern Ethiopia

**DOI:** 10.3389/fpubh.2022.1007308

**Published:** 2022-11-10

**Authors:** Habtamu Endashaw Hareru, Belay Negassa, Reta Kassa Abebe, Eden Ashenafi, Getachew Assefa Zenebe, Berhanu Gidisa Debela, Zemachu Ashuro, Negasa Eshete Soboksa

**Affiliations:** ^1^School of Public Health, College of Medicine and Health Science, Dilla University, Dilla, Ethiopia; ^2^Department of Environmental Health, College of Medicine and Health Science, Dilla University, Dilla, Ethiopia; ^3^Department of Reproductive Health, College of Medicine and Health Science, Dilla University, Dilla, Ethiopia

**Keywords:** associated factors, drivers, epidemiology, road traffic accidents, vehicles, risky driving behavior

## Abstract

**Background:**

Data on the magnitude of road traffic accidents (RTAs) were mostly obtained through police records and hospital registration data. However, insufficient data reporting masked the gravity of the problem, and little attention was paid to the magnitude and correlation of road traffic accidents from the driver's perspective. Therefore, this study aimed to assess the prevalence of RTA and related factors among drivers.

**Methods:**

A community-based cross-sectional study involving 316 drivers was conducted in Southern Ethiopia. The participants were chosen using a systematic random sample technique, and the data were obtained using an interview-administered structured questionnaire. To analyze the data, SPSS software (version 20) was employed. In addition to descriptive statistics, binary logistic regression analysis was also employed to find factors connected to traffic accidents. RTA factors were considered statistically significant if they had a *P*-value of 0.05 or below in the multivariate analysis.

**Result:**

The RTA among drivers was 126 (39.9%) (95% confidence interval (CI): 34.2–45.6%) in the previous year. The following factors were associated with RTA: vehicle maintenance (AOR = 0.11, 95% CI: 0.09, 0.96), media utilization (AOR = 0.38, 95% CI: 0.18, 0.65), participation in driving-related training (AOR = 0.73, 95% CI: 0.28, 0.91), punishment for prior traffic violations (AOR = 0.56, 95% CI: 0.47, 0.83), and risky driving behavior (AOR = 7.89, 95% CI: 3.22, 12.38).

**Conclusion:**

Two-fifths of the drivers were involved in a traffic accident. Risky driving behaviors, vehicle maintenance, media usage, attending driving-related training in the previous 2 years, and prior experience with traffic police punishment or warning were all strongly linked to road traffic accidents. In light of these statistics, the Federal Ministry of Transport of Ethiopia and other stakeholders should support making it mandatory for drivers to check their vehicles' safety, provide them with safety training, raise awareness about vehicle maintenance and risky driving behaviors, and enforce strict penalties for traffic violations.

## Introduction

A road traffic accident (RTA) refers to an incident on a public road or street that injures or kills one or more people and involves at least one vehicle in motion (1). As a major threat to public health and economic growth, RTA is a worldwide issue of grave importance. RTA is the eighth largest cause of mortality worldwide. The World Health Organization (WHO) expects RTAs to rise to become the fifth-greatest cause of mortality by 2030. These accidents are one of the top three causes of death for people aged 5 to 44 years and the leading cause of death for young people aged 5 to 29 years (2).

RTAs claim the lives of about 3,700 every day and roughly 1.35 million people each year. These RTA fatalities involved cars, buses, motorbikes, bicycles, trucks, and pedestrians. The high proportional burden is borne by pedestrians, cyclists, and motorcyclists, particularly those living in developing countries (3–5). Despite a minor decrease in road traffic deaths over the last decade, the current global fatality rate of 16.4 per 100,000 is staggeringly high (6). Around 50 million people are injured every year due to non-fatal injuries sustained in RTAs, posing a severe threat to global public health (2).

According to the WHO, despite having <60% of the world's registered vehicles, low and middle-income countries account for approximately 93% of deaths and 90% of disability-adjusted life years (DALYs) lost annually in road traffic crashes. Projections show that these figures will continue to rise over the next 20 years, with Africa having the highest fatality rate (5, 7).

An RTA is not only a tragedy that kills people but also a disaster that harms people, property, and the environment. The financial burden of these injuries falls heaviest on families, who must deal with both immediate and long-term costs, such as medical bills and lost savings. Road traffic injuries have a high financial impact nationally, especially in developing nations (7). Therefore, more effective control measures for the causes of road accidents are required if we are to achieve a reduction in road deaths and injuries by the year 2030 (8).

Economic losses due to road traffic fatalities and injuries are estimated to be 3% of global GDP and 5% of GDP in low- and middle-income nations (LMICs) (4). Africa has the highest rate of RTA fatalities per capita, with walkers and other vulnerable road users bearing the brunt of the burden. RTAs are now a major issue in Sub-Saharan Africa (SSA), accounting for roughly one-third of all regional deaths (9).

Despite the rising toll of RTAs, road safety is still a neglected concern in many developing countries. The health sector has been hesitant to acknowledge it as a priority public health concern (10, 11). A substantial amount of evidence implies that RTAs are easily avoided, and many high-income countries have successfully reduced the occurrence of these accidents through proven and cost-effective approaches (12). While there has been progress toward worldwide road safety metrics, it is sluggish, and RTA rates remain disproportionately high in LMICs (6). The WHO's global status report on road safety in 2018 predicts that SDG target 3.6 will not be met until 2020 (5). According to the Third Global Ministerial Conference on Road Safety in 2020, approximately 1.35 million people are killed or injured on the world's roads each year (13).

Moreover, in September 2020, the United Nations General Assembly declared the “Decade of Action for Road Safety 2021–2030,” with a new goal of a 50% reduction in RTA-related deaths and injuries by 2030, emphasizing the importance of a holistic approach to road safety and calling for continued improvements in road and vehicle design, as well as enhanced laws and law enforcement, and timely, life-saving emergency care for the injured (14). These alarming statistics highlight the importance of updating and enhancing road traffic accident data, which will aid policymakers in adopting evidence-based policies and programs for road safety.

In previous studies, the frequency of RTAs was reported to be 8% in Brazil (15), 30 % in Vietnam (16), 63.6% in another Brazilian study (17), 41.4% in an Indian study (18), and 51.50% in an Iranian study (19). RTA prevalence varies between sub-Saharan African studies, ranging from 74.0% in a study from Ghana to 87.5% in one from Nigeria (20, 21). In Ethiopian studies, the RTA varies from 23.5 to 62.5% (22–28).

According to a global status assessment on road safety, Ethiopia has at least 114 deaths per 10,000 vehicles per year, compared to only 10 in the United Kingdom and Ireland and 60 in 39 Sub-Saharan African nations (23). A number of studies have also indicated that Ethiopia has one of the world's highest fatality rates per vehicle (23, 24, 29–32). According to the World Health Organization (WHO) estimate, in 2013, the prevalence of road traffic fatalities in Ethiopia was 25.3 per 100,000 population, and the rate is among the highest in the world (25).

Although Ethiopia has a higher rate of road traffic morbidity and mortality, data on the scale of road traffic accidents were largely acquired through police records and hospital registration data. However, inadequate data reporting obscured the scale of the problem, and little concern was expressed (23, 30). Furthermore, even though road crashes are increasing, the government and other stakeholders have given little attention to the issue due to a lack of data on its magnitude and contributing factors from the driver's perspective. Therefore, the objective of this study was to determine the epidemiology of RTA and its associated factors among drivers in Dilla, Southern Ethiopia. Moreover, understanding drivers' perspectives on road traffic accidents can help in the development of policies and strategies for community-based intervention to reduce road traffic accident fatalities. In addition, it can also be used as a sole input to the literature.

## Materials and methods

### Study area and period

The study was conducted in Dilla Town, Gedeo Zone, Southern Ethiopia. Dilla town is one of the two city administrations established in 1904 in the Gedeo Zone. It is 377 km from the capital city, Addis Ababa, and has one main road passing through the town from Addis Ababa to Moyale. The total population in Dilla is 94,189; of which 46,058 (49.9%) are males, and 48,131 (50.1%) are females. During the data collection period, the estimated number of vehicles serving public transportation in Dilla town was 1,266. Bajaj 502, taxis 15, and 159 are private cars (automobiles), 85 are government vehicles, and 505 are motorcycles. There were 304 registered and 673 unregistered vehicles (Source: Dilla Town Administrative Road Transport Office). The study was conducted from 18 October 2021, to 20 November 2021.

### Study design

A community-based cross-sectional study design was conducted to assess the epidemiology of road traffic accidents and associated factors among drivers in Dilla Town, Southern Ethiopia.

### Population

The survey included everyone in Dilla town who drove a privately or government-owned vehicle (automobile, taxi, or Bajaj). All drivers of the selected vehicles outside Dilla, those with <12 months of driving experience before the data collection period, and those who were either unwell or unavailable throughout the study period were excluded.

### Sample size determination

The sample size was calculated using the single-population proportion formula, with prevalence estimates from previous research conducted in Adama city of 56.4 % (27), a margin of error of 5%, and a two-sided 95% confidence interval (Zα/2 = 1.96). After attaining the correction formula because the total number of vehicles was <10,000 and by including a 10% non-response rate, the appropriate sample size for this study was 319.

### Sampling techniques

During the study period, there were 1,266 vehicles in the town, of which 15,502, 159, 85, and 505 were taxis, Bajaj, private cars, automobiles, governmental cars, and motorcycles, respectively. A sampling frame of all cars was received from the Dilla Town Road and Transport Bureau, and the computed sample size was proportionally allocated. Individual drivers were also selected using systematic random sampling using their board numbers.

### Study variables

The dependent variable in the study was a road traffic accident. The independent variables included socio-demographic variables (sex, age, marital status, educational status, family size, monthly income, and additional job), personnel (drivers) behavior (chewing khat, habits of alcohol drinking, talking on mobile phones while driving, and driving at speed), vehicle-related factors (service year of the vehicle (perceived vehicle age, vehicle maintenance, and frequency of vehicle health check), and failed brake or mechanical road-related conditions (congested, high traffic roads, damaged roads, and others), driving-related training in the past 2 years, years of holding a driving license, any prior violation of traffic rules, mass media utilization, perceived availability of sufficient traffic signs, and driving experience.

### Operational definition

#### Road traffic accident

In this study, a road traffic accident is defined as a collision between cars, between vehicles and pedestrians, between vehicles and animals, or between vehicles and a fixed object that occurred within the previous 12 months. The questions were close-ended. If the driver self-reported RTA experience at least once in the last 12 months, the answer was a “yes.” Otherwise, the answer was a “no.”

#### Risky driving behavior

If a respondent had engaged in any of the four risky driving behaviors in the last 12 months (i.e., driving after drinking alcohol, chewing khat, driving at high speeds, mobile phoning, or receiving while driving), the answer was a “yes.” Otherwise, the answer was a “no.”

### Data collection instrument and procedure

An interview-administered structured questionnaire was used to collect data related to the study objectives. Questions about road traffic accidents and related factors were adapted from previous research (27, 32–37). The data collection tools contained four sections: the first section included the socio-demographic characteristics of the participants; the second section identified the magnitude of road traffic accidents and risky driving behavior of the participants; the third section assessed vehicle-related factors; and the fourth section assessed road-related conditions. Fourth-year public health officers collected the data. After explaining the goal of the study, the interviewers approached the selected study participants at their station for Bajaj and taxi drivers, at their residence or fuel station for automobile drivers, and at their institution for government office cars using information obtained from the city's road and transport office.

### Data quality control

To assure the quality of the data, the questionnaire that was primarily written in English was translated into the local language (Gede'ogna) and then translated back to ensure that it was translated accurately. 1 week before the actual data collection, the instrument was pretested on 5% of drivers from areas other than the research area (Wonago town), and the questionnaire was modified accordingly. 2 days of training were given to the data collectors and supervisors about the research question, sampling technique, data handling, ethical conduct, and quality of data collection. The questionnaires were checked for accuracy and completeness every day by the primary investigator, and modifications were made if errors were discovered in the collected data.

### Data processing and analysis

The data were entered into EPI Info version 7 and analyzed using SPSS version 20 statistical software. Categorical variables were described using frequency or percentage, whereas continuous variables were described using mean and standard deviation. Binary logistic regression analyses were used to determine the influence of each independent variable on road traffic accidents. Those variables with a *p*-value of < 0.25 on bivariate analysis were chosen for multivariate analysis. Variables with a *p*-value of < 0.05 in the multivariate analysis were considered significant factors linked with road traffic accidents. Finally, the adjusted odds ratio (AOR) and 95% confidence interval (CI) for statistically significant variables were computed and interpreted.

### Ethical consideration

Ethical approval was secured from the Institutional Review Board of Dilla University (IRB-DU), College of Medicine and Health Science. Permission letters were also secured from Dilla town road and transport offices. Before data collection, information about the purpose of the study and the right not to participate was given to the participants. Oral consent was obtained from all the study participants, and the information collected from the participants was kept confidential.

## Results

### Socio-demographic characteristics

In the current study, out of 319 participants, 316 were included, with a 99% response rate. The majority of the participants (94.6%) were males, with a mean standard deviation (SD) age of 31 (12.6) years and 174 (55%) in the age group of greater than 31 years. More than half (52.2%) of the participants were not married, 142 (45%) had secondary educational status, and 243 (76.9%) had no additional work (Table 1).

**Table 1:** Socio-demographic characteristics of the participants in Dilla Town, Gedeo Zone, Southern Ethiopia, 2021 (n = 316).

**Variables**		**Frequency**	**Percent (%)**
Age (in years)	<31	142	45%
	≥31	174	55%
Sex	Male	299	94.6
	Female	17	5.4
Educational status	No formal education	19	6
	Primary	77	24.4
	Secondary	142	45
	College and above	78	24.6
Marital status	Not married	165	52.2
	Married	151	47.2
Family size (number)	≤ 5	101	32.0
	>5	215	68.0
Monthly income (ETB)	<1,000	26	8.2
	1,000–2,000	107	33.9
	2,000–3,000	100	31.6
	>3,000	83	26.3
Any additional work	Yes	73	23.1
	No	243	76.9

ETB, Ethiopian Birr.

### Vehicle and driver related variables

Of 316 respondents, ~124 (39.2%) were driving their own car. Nearly 80% of the vehicles were Bajaj and motor bicycles. The majority of the respondents (64.6%) had between 1 and 4 years of driving experience. Of these, 201 (63.6%) had had the license for 1 to 4 years. Over half of the participants received drivers related training in the last 2 years.

Moreover, 182 (57.6%) of the participants reported that their vehicles were served for about 1–4 years, and 216 (68.4%) of the participants informed us that their vehicles were checked every 6 months. About three-fourths (75.9%) of the participants also reported that their vehicles had some kind of service in the last year (Table 2).

**Table 2:** Drivers and Vehicle-related characteristics of the participants in Dilla Town, Gedeo Zone, Southern Ethiopia, 2021 (n = 316).

**Variables**		**Frequency**	**Percentage (%)**
Vehicle type	Bajaj	125	39.6
	Motor bicycle	125	39.6
	Taxi	4	1.3
	Minibus	32	10.1
	Mid bus	8	2.5
	Public service	22	7
	Total	316	100
Driver relation with the car	Owner	124	39.2
	Recruited	128	40.5
	Relative to owner	41	13
	Governmental	20	6.3
	Other	3	0.9
	Total	316	100
The driving experience of the drivers	<1 year	64	20.3
	1–4 years	204	64.6
	5–10 years	48	15.1
	Total	316	100
Years of holding a driving license	<1year	39	12.3
	1–4 years	201	63.6
	4–10 years	70	22.2
	Above 10 years	6	1.9
	Total	316	100
Presence of sufficient zebra line	Yes	54	20.7
	No	262	82.3
	Total	316	100
Have you attained any drivers related training in the last 2 years	Yes	165	52.2
	No	151	47.8
	Total	316	100
For how long has this vehicle served? (in years)	<1	67	21.2
	1–4	182	57.6
	5–10	63	19.9
	>10	4	1.3
	Total	316	100
Any kind of service in the last year for your vehicles	Yes	272	86.1
	No	44	13.9
	Total	316	100
How frequently do your vehicle healthy checked?	Every 6 month	216	68.4
	Every year	73	23.1
	Every 2 years	19	6.0
	≥3 years	8	2.5
	Total	316	100
Any kind of service in the last year for your vehicles	Yes	240	75.9
	No	76	24.1
	Total	316	100
Working hours	8 h and less	107	33.9
	More than 8 h	209	66.1
	Total	316	100
Did you have a habit of following mass media	Yes	140	44.3%
	No	176	55.7%
	Total	316	100
Perceived availability of sufficient traffic signs or symbol	Yes	81	25.6%
	No	235	74.4%
	Total	316	100
Perceived road condition	Good	43	13.6%
	Poor	273	86.4%
	Total	316	100

### Risky driving behaviors of the participants

**Figure 1:**
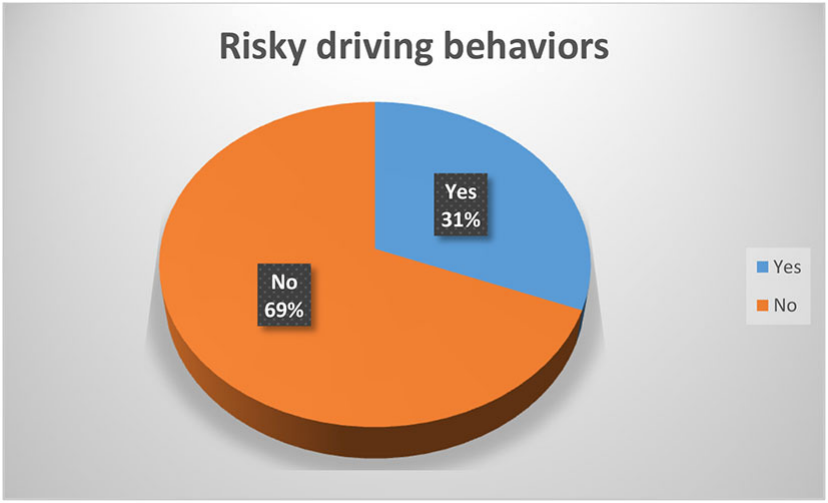
Risky driving behaviors among drivers in Dilla Town, Gedeo Zone, Southern Ethiopia, 2021 (n = 316).

A respondent's risky driving behavior was evaluated when they engaged in one of four behaviors: driving after consuming alcohol, chatting on a cell phone while driving, chewing khat while driving, and/or driving at high speeds. A total of 98 (31.0 %) of the 316 drivers in this study engaged in risky driving behaviors (Figure 1). Driving at high speeds, chewing khat while driving, driving after drinking, and the habit of talking on the phone while driving accounted for 24.5, 35.7, 13.3, and 26.5% of the 98 risky driving behaviors, respectively.

### The magnitude of road traffic accidents

**Figure 2:**
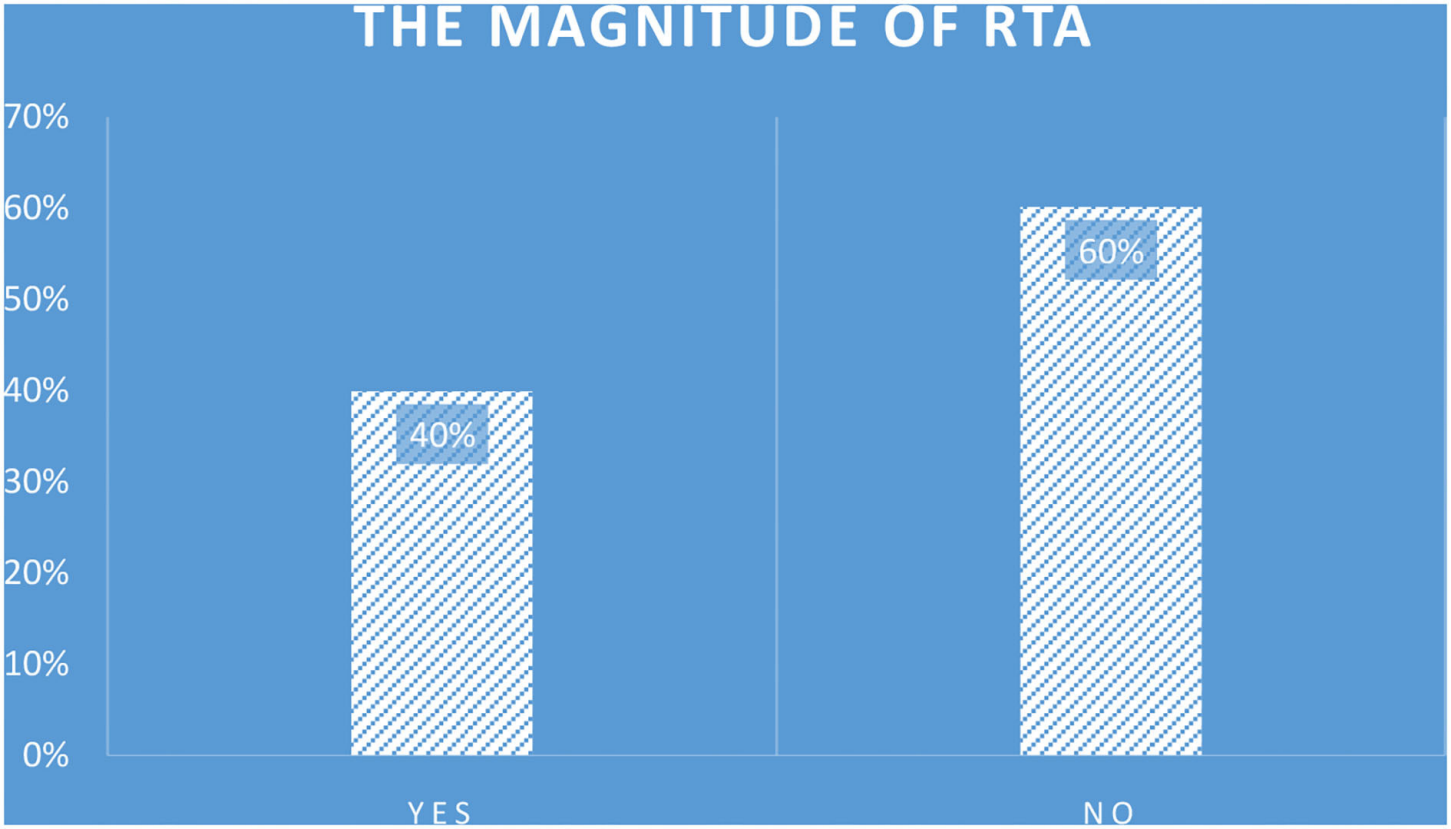
The magnitude of road traffic accidents among drivers in Dilla Town, Gedeo Zone, Southern Ethiopia, 2021 (n = 316).

The magnitude of RTA among drivers in the past year in Dilla Town was 126 (39.9%) [95% confidence interval (CI): 34.2–45.6%] (Figure 2).

**Figure 3:**
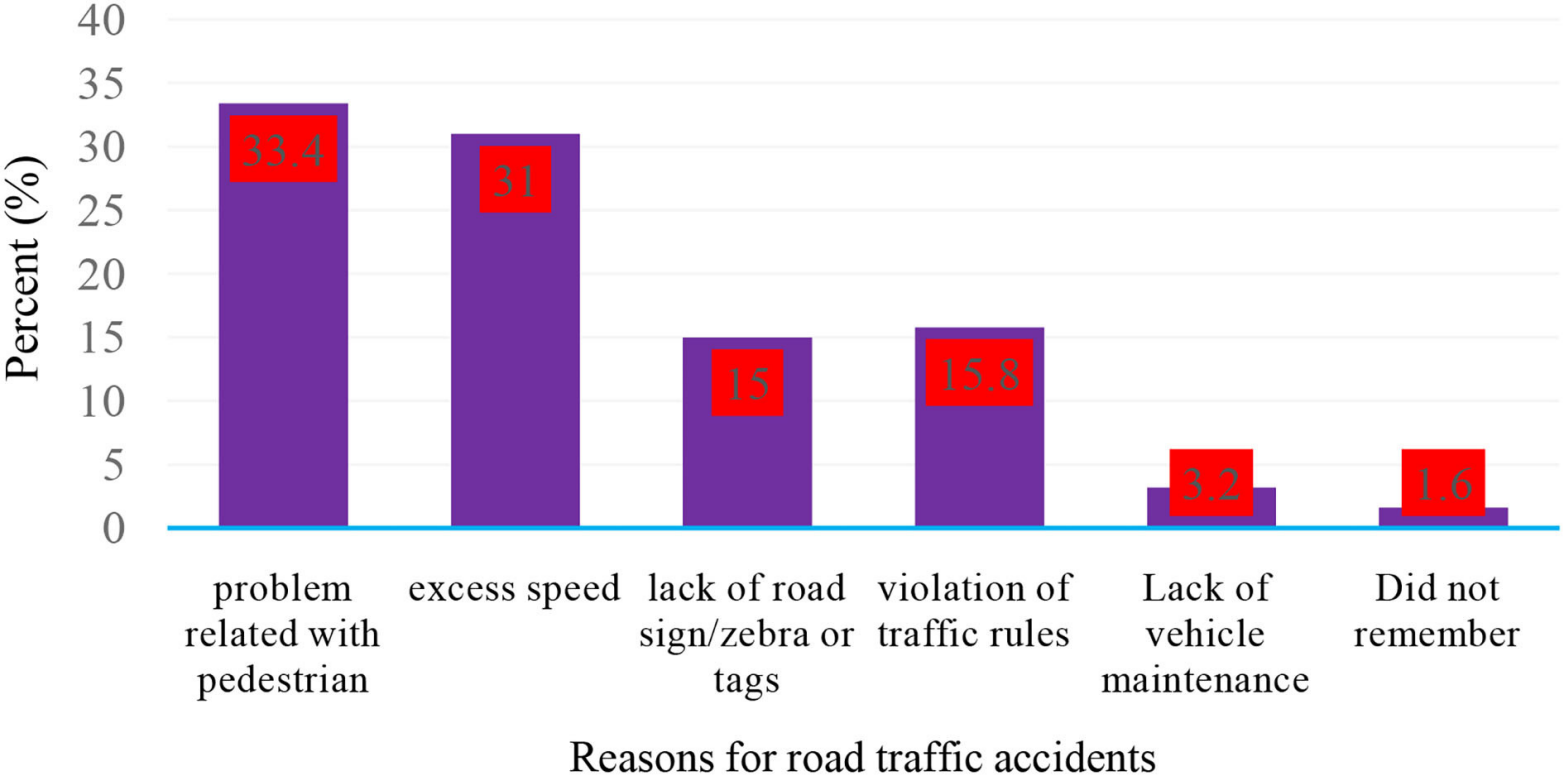
Reasons for road traffic accidents among participants who have had road traffic accidents in the past year in Dilla Town, Gedeo Zone, Southern Ethiopia, 2021 (n = 316).

A total of 42 (33.3%) of 126 participants who had an RTA cited pedestrian concerns as the leading cause of incidents that occurred during the previous 12 months; 39 (30.9%) cited excessive speed; 19 (15%) cited a lack of road signs/zebras or tags; and 20 (15%) cited a breach of traffic rules (Figure 3).

### Factors associated with road traffic accidents

Variables that have a *p*-value of < 0.25 in the bivariate logistic regression analysis and are eligible for multivariate logistic regression analysis include gender, educational status, additional job, average monthly income, driving experience, listening to mass media, attending recent driving-related training, receiving any kind of service to a vehicle in the past year, prior punishment by traffic police, availability of sufficient traffic signs or symbols, and risky driving behavior.

Finally, independent variables significantly associated with road traffic accidents at *p*-value < 0.05 in multivariate logistic regression were those who received any kind of service to a vehicle in the past year, listened to mass media, received driving-related training in the last 2 years, prior punishment by traffic police; and risky driving behavior.

RTA risk was 89 % lower in vehicles that had maintenance in the previous year than in vehicles that did not [AOR = 0.11, 95% CI: 0.09, 0.96, *P*-value = 0.001]. Drivers who had received driving-related training in the previous 2 years were 23% less likely to be involved in an RTA than those who did not [AOR= 0.73, 95%CI: 0.28, 0.91, *P*-value = 0.033]. Drivers who had been charged or warned by traffic cops in the previous year were 44% less likely to have RTA than those who had not [AOR = 0.56, 95% CI: 0.47, 0.83, *P*-value = 0.041]. RTA was 62 % less likely among drivers who could follow mass media than among those who could not [AOR = 0.38, 95% CI: 0.18, 0.65, *P*-value = 0.003].

Moreover, drivers who engaged in risky driving behaviors were nearly eight times more likely to be involved in an RTA than those who did not [AOR = 7.89, 95%CI: 3.22, 12.38, *P*-value = 0.029] (Table 3).

**Table 3:** Factors associated with road traffic accidents among vehicle drivers in Dilla Town, Gedeo Zone, Southern Ethiopia, 2021 (n = 316).

**Variables**	**Prevalence of RTA**		**COR [95% CI]**	**AOR [95%CI]**	***P*-value**
	**Yes**	**No**			
**Age (years)**					
<31 ≥31	62 64	80 110	1.33 [0.85, 2.09] 1		
**Sex**					
Male Female	119 7	180 10	0.94 [0.35, 2.55] 1	0.63 [0.69, 4.55] 1	0.89
**Educational status**					
No formal education Primary school Secondary school College and above	8 37 56 25	11 40 86 53	1.54 [0.55, 4.31] 1.96 [1.02, 3.77] 1.38 [0.77, 2.47] 1	1.14 [0.23, 2.33] 2.34 [0.79, 4.67] 1.61 [0.55, 3.42] 1	0.07 0.09 0.17
**Marital status**					
Not married Married	69 57	96 94	1.96 [0.75, 1.86] 1		
**Family size**					
Less than or equal to 5 Greater than 5	45 81	56 134	0.75 [0.46,1.21] 1		
**Additional job(s)**					
Yes No	26 100	47 143	0.79 [0.46, 1.36] 1	2.45 [0.49, 2.38] 1	0.628
**Average monthly income**					
Less than 1,000 1,000–2,000 2,000–3,000 Greater than 3,000	7 32 42 45	19 62 54 55	2.22 [0.86,5.75] 1.58 [0.88,2.83] 1.0 5 [0.59,1.85] 1	1.11 [0.92,6.65] 4.56 [0.13,8.34] 2.09 [0.93,7.34] 1	0.667 0.083 0.467
**Did you listen to mass media**					
Yes No	31 95	109 81	[0.15, 0.41] 1	0.38 [0.18,0.65] 1	0.003**
**Vehicle maintenance**					
Yes No	21 105	55 135	0.49 [0.28,0.86]* 1	0.11 [0.09,0.96]* 1	0.001**
**Driving experience**					
Less than one year 1–4 years 5–10 years	45 60 21	19 144 27	3.05 [1.39,6.66] 0.54 [0.28,1.02] 1	4.81 [0.89,7.86] 0.78 [0.14, 2.61] 1	0.178 0.073
**Working hours**					
8 h and less Greater than 8 h	42 84	65 125	0.96 [0.60, 1.55] 1		
**Attending driving-related training in the last 2 years**					
Yes No	38 88	127 63	0.21 [0.13, 0.35]* 1	0.73 [0.28, 0.91] 1	0.033**
**Presence of sufficient zebra line**					
Yes No	21 105	33 157	0.95 [0.52, 1.73] 1	0.58 [0.22, 1.50] 1	0.289
**Prior punishment by traffic polices**					
Yes No	30 96	91 99	0.34 [0.21, 0.56]* 1	0.56 [0.47, 0.83] 1	0.041**
**Perceived road condition**					
Good Poor	16 110	27 163	0.88 [0.45, 1.71] 1	0.53 [0.19, 1.47] 1	0.09
**Availability of sufficient traffic signs or symbol**					
Yes No	27 99	54 136	0.69 [0.40, 1.17]* 1	0.45 [0.98,3.07] 1	0.065
**Risky driving behaviors**					
Yes	75	23	10.68 [6.08, 18.74]	7.89 [3.22, 12.38]	0.029*
No	51	167	1	1	

AOR, Adjusted Odds Ratio; COR, Crude Odds Ratio; *p-value < 0.25 in the bivariate analysis; **p-value ≤ 0.05 in the multivariate analysis; Not married- Single, widowed, and divorced.

## Discussion

In this study, the magnitude of road traffic accidents (RTA) among drivers in the past year in Dilla Town was 126 (39.9%) (95% CI: 34.2–45.6%). This finding is consistent with previous studies conducted in India, in which 41.4% of road traffic accidents occurred among drivers (18). Another Ethiopian study conducted in Amhara National Regional State reported 34.5% of RTA among passenger vehicles (23). However, this study's finding is not consistent with the study conducted among motorcycle taxi drivers in 32 regions of Caicó; the Rio Grande do Norte, Brazil, which found that 63.6% of them were involved in at least one motorcycle accident (17). In Southern Nigeria, (87.5%) of commercial motorcyclists were involved in road traffic accidents the year before the study (21). In Ghana, 74.0% were reported to have been involved in crashes in the past year before the study (20). A systematic review, GIS, and meta-analysis study in Iran showed that the prevalence of road traffic accidents was 51.50% (19), and a study was conducted in Adama (54.7%) (29). Moreover, it is not consistent with a hospital-based study conducted in Wolaita Zone, SNNPR, Ethiopia, in which 62.5% of trauma victims were due to road traffic accidents (30). This might be due to the underreporting of accidents in the study settings.

The prevalence rate found by this study was also higher than that in a study conducted in Brazil, where the prevalence of road traffic accidents among motorcyclists in the previous 12 months was 8% (15). Studies conducted in Vietnam showed that 22.7% and 30% of RTA occurred among taxi drivers and motorcyclists over 1 year, respectively (16, 31, 38); in Mekelle, Ethiopia, 26.4% of taxi drivers reported involvement in a road traffic crash within the past 3 years (32). A study conducted in Chuko town, Sidama, Ethiopia, reported a lifetime prevalence of RTA of 23.55% (26). In another study conducted in Mekelle, Ethiopia, where 26.4 % of taxi drivers reported involvement in a road traffic crash over the preceding 3 years (39), a study conducted in East Wollega, Western Ethiopia, reported a lifetime prevalence of RTA of 33%

[31], and a similar study conducted in Jigjiga, Ethiopia discovered the prevalence of RTA in the previous three years was 32.8% (27). The main differences between current research and previous studies in road traffic accidents could be attributed to a lack of safety knowledge; risky driving practices by drivers, such as drinking alcohol and chewing khat while driving (33); differences in the study period; the recent increase in the number of vehicles; inadequately experienced drivers; and the approval from drivers who were not adequately trained. Additional factors that may contribute to the high rate of RTA in the research area include a lack of formal training, mobile phone use while driving, dense traffic, failure to prioritize pedestrians, and a lack of awareness campaigns on road safety regulations. Sixty-six percent of drivers in the research setting had more than 8 h of work each day, suggesting that fatigue (which impairs decision-making) from working long hours may be linked to the high rate of road traffic accidents observed.

According to this study, drivers who follow social media are less likely to be involved in a car accident than those who do not. This study was consistent with a previous study conducted in Mekelle town, northern Ethiopia (32). Therefore, research done in India and Kenya found that media coverage of road traffic accidents can be used as a preventive measure by documenting the experiences of those hurt in RTAs and informing evidence-based solutions; emphasizing the systems approach, which encourages government action (34) and encourages people to share information and opinions about road safety through social media (35), which can change individual behavior by raising awareness and increasing understanding of the issue.

This study found that prior traffic police punishment was less likely to be associated with road traffic accidents among taxi drivers, which is consistent with previous research conducted in Mekelle, northern Ethiopia (32), Hanoi, Vietnam (36), and India (37), which found that improper enforcement of rules and regulations pertaining to road traffic causes accidents. Penalties for traffic violations may thereby reduce the risk of traffic violations and accidents by motivating drivers to observe traffic laws and improve their understanding of road safety. As a result, both the recent and earlier studies may have implications for altering the kind, degree, and scope of enforcement of traffic violation penalties used generally in Ethiopia, as well as the study's specific context. This may not always be the case. According to a study from China, driving experience significantly impacts how frequently violations occur and accidents happen (40).

The prevalence of RTA in this study was lower in vehicles that had been maintained in the preceding year than in vehicles that had not, which is similar to a study conducted in Ghana (41) and Nigeria (42). Because of this, well-maintained cars not only have a longer lifespan but also improve road safety for all parties involved, including drivers, passengers, and other road users. Accidents and injuries can result from failing to keep a vehicle in excellent operating condition, and a well-designed and maintained vehicle is less likely to be involved in accidents.

According to the current study, drivers who received driving-related training in the previous 2 years were 23 % less likely to be involved in an RTA than those who did not. A study on the efficacy of pre-and post-license driver training found that post-license training programs help improve skills considered relevant to crash prevention, such as hazard perception and advanced vehicle control skills (43). Therefore, it is recommended that drivers receive the necessary driving-related training to improve their skills and learn about safety measures to prevent traffic accidents and behavioral change in drivers toward risky driving behaviors. Training also enables drivers who had insufficient training during their license examinations to obtain the necessary knowledge.

Moreover, drivers who engaged in risky driving behaviors were more likely to be involved in an RTA than those who did not. This finding is supported by research conducted in Mekelle City, where drivers who consumed alcohol were more likely to be involved in an RTA than those who did not (39), as well as a study conducted in Pakistan, Korea (38), and Tehran, Iran (44). In addition, a study conducted in Hanoi, Vietnam, found that using a cell phone while riding in a taxi was linked with road traffic accidents (45). As a result, to successfully minimize the magnitude of road traffic incidents, a proper countermeasure against drivers who engage in risky driving behaviors should be devised. Furthermore, to reduce road traffic accidents, alcohol intake tests, substance use, driver belt use, driver health status, driving speed, and vehicle maintenance status should be checked regularly.

### Limitations of the study

The study's limitations were that taxi drivers are a highly mobile community, which can contribute to discrepancies in the selection of study participants. The study only included living drivers, which can lead to an underestimation of the significance of the conclusion. Underestimation of the prevalence could also be due to social desirability bias. The current study also failed to evaluate drivers' perceptions of risky behavior and other experiences with it. Additionally, some demographic data were not collected, including height, weight, and BMI, which could be used to assess the risk of obstructive sleep apnea and determine whether a participant was at fault or injured in an accident.

## Conclusion

In this study, two-fifths of the drivers in Dilla town had a traffic accident in the previous year (2020) preceding the data collection period. Factors such as the experience of punishment or warning by traffic police in the last year, vehicle servicing, listening to mass media, attending driving-related training in the previous 2 years, and risky driving behaviors were all significantly associated with road traffic accidents. As a result, the Federal Ministry of Transport of Ethiopia and other stakeholders should be encouraged to advise drivers on vehicle safety and driving-related safety training, enhance their capacity to listen to the media, and raise awareness about vehicle maintenance and unsafe driving behaviors. Traffic officials should inspect the safety of the vehicles and advise all car owners to verify their vehicles' service. Additionally, it is recommended that drivers who disobey traffic laws and exhibit unsafe driving behaviors should be made aware of their actions and punished. Additional prospective and qualitative research is required to provide detailed accident characteristics and taxi driver behavior and perception.

## Data availability statement

The original contributions presented in the study are included in the article/supplementary material, further inquiries can be directed to the corresponding author/s.

## Ethics statement

The studies involving human participants were reviewed and approved by the Institutional Review Board of Dilla University (IRB-DU), College of Medicine and Health Science. The patients/participants provided their written informed consent to participate in this study.

## Author contributions

HH and BN were involved in the conceptualization, design, analysis, interpretation, and drafting of the manuscript and the critical revision of intellectual content. RK, EA, GZ, ZA, and NE were involved in the study's design, data analysis, data interpretation, and manuscript drafting and revision. All authors agree to be accountable for all aspects of the work and have approved the final version of the manuscript to be published.

## Conflict of interest

The authors declare that the research was conducted in the absence of any commercial or financial relationships that could be construed as a potential conflict of interest. The reviewer CK declared a shared affiliation with the author(s) to the handling editor at the time of review.

## Publisher's note

All claims expressed in this article are solely those of the authors and do not necessarily represent those of their affiliated organizations, or those of the publisher, the editors and the reviewers. Any product that may be evaluated in this article, or claim that may be made by its manufacturer, is not guaranteed or endorsed by the publisher.

## Abbreviations

AOR: Adjusted Odd Ratio
CI: confidence interval
DALY: disability-adjusted life years
GDP: gross domestic product
IRB: institutional review board
LMIC: lower- and middle-income countries
RTA: road traffic accidents
SNNPR: South Nation Nationalities of People Republic
WHO: the World Health Organization.
